# Effect of treatment variables on apical extrusion of debris during root canal retreatment: A systematic review and meta-analysis of laboratory studies

**DOI:** 10.34172/joddd.40501

**Published:** 2024-03-29

**Authors:** Emel Uzunoğlu Özyürek, Selen Küçükkaya Eren, Sevilay Karahan

**Affiliations:** Department of Endodontics, Faculty of Dentistry, Hacettepe University, Ankara, Turkey

**Keywords:** Endodontics, Root canal, Retreatment

## Abstract

**Background.:**

This study aimed to systematically and comprehensively review the effect of various treatment variables on apically extruded debris (AED) during non-surgical root canal retreatment (NSRCRT).

**Methods.:**

The study protocol is shared in the Open Science Framework database (https://osf.io/kjtdg/?view_only=17060180705745ec9dae9a01614f3880). An electronic search was conducted up to July 2022 to reveal related studies. Two reviewers critically assessed the studies for eligibility against inclusion and exclusion criteria and data extraction. Quantitative data synthesis was performed, and the risk of bias in the studies was also evaluated.

**Results.:**

Forty-six studies were included in the systematic review and 14 in the meta-analysis. Conflicting or limited evidence was found for the effect of sealer type, obturation technique, and solvent use. The manual instrumentation increased the amount of AED compared to rotary instrumentation during the removal of filling materials (*P*<0.001). There was no significant difference in the amount of AED between the use of rotary and reciprocating files during the removal of filling materials (*P*=0.181).

**Conclusion.:**

Rotary instruments can be recommended instead of manual instruments during the removal of filling materials to control the amount of AED. Further studies with a low risk of bias are needed to clarify the effect of other treatment variables on AED during NSRCRT.

## Introduction

 The success of primary root canal treatment is reported to be high; however, in case of failure, non-surgical root canal retreatment (NSRCRT) is the first treatment option for the survival of the tooth.^1^ The complete removal of root canal filling materials and thorough cleaning of the root canal system are important factors for the success of NSRCRT. It has been reported that the remaining filling materials in the root canal could be the reason for persistent infections.^2,3^ Some instrumentation systems, irrigants, and irrigation techniques have been used to enhance the cleaning of the root canal system during NSRCRT.^4-6^ Stainless steel hand files, rotating and reciprocating nickel-titanium (NiTi) instruments, and ultrasonic tips have been used to efficiently clean and shape the root canals during NSRCRT procedures.^4,7-9^ Solvents such as chloroform, xylol, halothane, and orange oil can be used to remove previous filling materials from the root canal system.^7,10-12^ Several irrigants^13^ and various irrigant activation techniques^14^ have been further used to improve the cleanliness of root canal walls during NSRCRT. While the main goal is to obtain a clean root canal system as much as possible, apical extrusion of debris containing dentin chips, microorganisms, necrotic tissue remnants, and previous filling materials is also a major concern during NSRCRT.^15-17^ It has been reported that apically extruded debris (AED) may cause postoperative pain^15,18-20^ and swelling and may impair periapical healing.^2,3,15,21,22^

 The effects of treatment variables on AED at preparation, irrigation, or obturation of root canals during NSRCRT have been widely studied in the literature.^7,23-26^ It has been reported that all instrumentation systems have the potential for apical debris extrusion during NSRCRT.^23,27,28^ The root canal preparation with hand files has been reported to extrude more debris apically compared to rotary and/or reciprocating systems.^28-30^ On the other hand, other studies reported no difference in the amount of AED between manual instrumentation and engine-driven instrumentation (rotary, reciprocating, or ultrasonic instrumentation).^4,31^ There is also no consensus in studies comparing the effect of rotary and reciprocating instruments on AED.^8,23,25,32-35^ Some studies have reported no difference between these instruments,^4,35,36^ while others have reported that rotary instruments extruded apically more debris compared to reciprocating instruments^37,38^ or vice versa.^32^ In addition to the instrumentation methods, other treatment variables, such as materials or techniques used to remove filling materials and obturation of the root canal system, may also influence the amount of AED.^11,12,39-41^ Clarifying the parameters that may affect the apical extrusion of debris during NSRCRT may contribute to clinical practice. Therefore, this study aimed to systematically and comprehensively review the effect of treatment variables during NSRCRT on the apical extrusion of debris.

## Methods

###  Search strategy

 PROSPERO registration could not be performed because of including in vitro studies; therefore, the study protocol is available online in the Open Science Framework database (https://osf.io/kjtdg/?view_only=17060180705745ec9dae9a01614f3880). Preferred Reporting Items for Systematic Reviews and Meta-Analyses (PRISMA) guidelines were followed for a literature search.^42^ The study protocol is shared. A comprehensive search was conducted with Cochrane Library, Google Scholar, Lilacs, PubMed, Scopus, Web of Science, and Open Grey databases to reveal related English-language studies up to July 2022. The interest of this review was to reveal the influence of any step used during the entire NSRCRT procedure on the amount of AED. The population, intervention, comparison, and outcome (PICO) strategy was used for the structured review question:

1. Population: Extracted mature permanent human teeth obturated with gutta-percha and undergoing NSRCRT procedure
2. Intervention: Any variable in the obturation phase or retreatment phase during the treatment of samples
3. Comparison: Any variable in the obturation phase or retreatment phase during the treatment of samples
4. Outcome: The effect of the tested treatment variable on the amount of AED

The search terms were extrusion, extruded, debris, gutta-percha, gutta-percha, sealer, sealant, filling material, retreatment, endodontic, and root canal. These keywords were combined as (((extrusion or extruded) AND (debris or gutta-percha or gutta percha or sealer or sealant or filling material)) AND (retreatment)) AND (endodontic or root canal). Articles published in the Australian Endodontic Journal, Journal of Endodontics, and International Endodontic Journal were checked during keyword selection. Modifications were performed in each database according to their search tools. Supplementary Table 1 reveals examples of the search strategy of databases. Reference lists of all the included articles were manually searched through an electronic search for additional articles that were not identified.

###  Screening and selecting studies

 Initially, an electronic search was conducted by two reviewers independently to find relevant articles by title. Then, the abstracts of all potential articles were attentively checked to detect eligible studies. When the data obtained through title and abstract screening were insufficient, the full text of the article was read during selection.

 Studies were selected for inclusion if they fulfilled the following criteria:

1. Laboratory studies on fully formed human teeth, using gutta-percha as the main root canal obturating material
2. Studies testing the effect of at least one parameter either in the obturation step (the type of root canal sealer used, the type of obturation technique applied, etc) or in the NSRCRT (the type of instrument used, the type of instrumentation technique applied, the use of solvent, etc)
3. Studies comparing the weight of dry AED following the NSRCRT of teeth
4. Studies in the English language

Inclusion was based on consensus between the two reviewers. Studies using different core materials than gutta-percha during obturation, studies in a different language than English, studies using immature teeth or resin blocks, studies reporting AED as volume/area or as the score after visual observation, studies comparing the effect of different brands of root canal instruments operating in the same type of motion kinetic that were not manufactured specifically for retreatment or studies using different debris collectors than empty pre-weighted tubes/vials such as paper filters, aluminum crowns, and aluminum foils, studies evaluating only the cytotoxicity of AED, and studies measuring the weight of AED without performing the drying step were excluded from the present review.

###  Data extraction

 The full texts of all the included studies were obtained, and a standardized form was used by two reviewers during data extraction. The extracted variables were ethics approval, tooth type, root curvature, the determination of working length (WL), the final file used before obturation, obturation technique, filling materials, incubation period following obturation, debris collection method, periodontal ligament simulation, instruments used for retreatment, the last file used at the WL in the NSRCRT, solvent use during retreatment, irrigants used during retreatment, patency control after retreatment, including the debris around the outer surface of the root in the measurement, storage condition of debris collectors following NSRCRT, the statistical analysis method used, conflict of interests, and main outcomes.

###  Risk of bias evaluation

 Quality assessment of the included studies was performed by the risk of bias analysis. Previous studies were considered during the risk of bias evaluation.^43,44^ The following parameters were assessed: sample size calculation, randomization of teeth, standardization of samples based on root canal shape, standardization of samples based on apical diameter, confirmation of the quality of root canal obturation before proceeding to NSRCRT procedures, preparation of samples by a single operator and/or experienced operator, the use of retreatment instruments according to the manufacturers’ instructions, blinding of the operator during NSRCRT, standardization of irrigant volume used during NSRCRT for each group, and confirmation of filling materials’ removal following NSRCRT. If the parameter was reported in the article, it received a Y (yes); if it could not be found in the article, it received an N (no). According to the Y numbers, the bias risk of the article was classified as high (1–4 Yes), medium (5–7 Yes) and low (8‒10 Yes). Articles were checked independently again by two reviewers, and, in case of controversy, the articles were re-assessed together by the reviewers. Missing data were requested from the corresponding authors via e-mails at least twice.

 An evidence synthesis was carried out as follows.^43,44^

1. Strong evidence: When two or more studies with a low risk of bias and ≥ 75% of the studies reported consistent findings
2. Moderate evidence: When one study with a low risk of bias and/or two or more studies with a medium or high risk of bias reported consistent findings.
3. Limited evidence: When only one study with a medium or high risk of bias provided results.
4. Conflicting evidence: When studies provided inconsistent results (consistent findings were reported by < 75% of the studies).
5. No evidence: When no study could be found.

###  Meta-analysis

 Quantitative data synthesis of the included studies was performed to combine comparable results using a software program for meta-analysis (MedCalc Statistical Software trial version 19.0.5, MedCalc Software, Ostend, Belgium). The weight of AED was selected as the outcome. The number of specimens in each group and the mean and standard deviation related to respective comparisons were extracted from the studies. Standardized mean difference (SMD) was calculated for each study included in the meta-analysis. Statistical heterogeneity between studies was analyzed using the I^2^ value, showing low, medium, and high heterogeneity at 25%, 50%, and 75%, respectively.^45^ Fixed-effects models were used when I^2^ scores were toward 0%; when I^2^ scores were toward 100%, random-effects models were used. Forest plots were used to show the results of all analyses.

## Results

**Figure 1:**
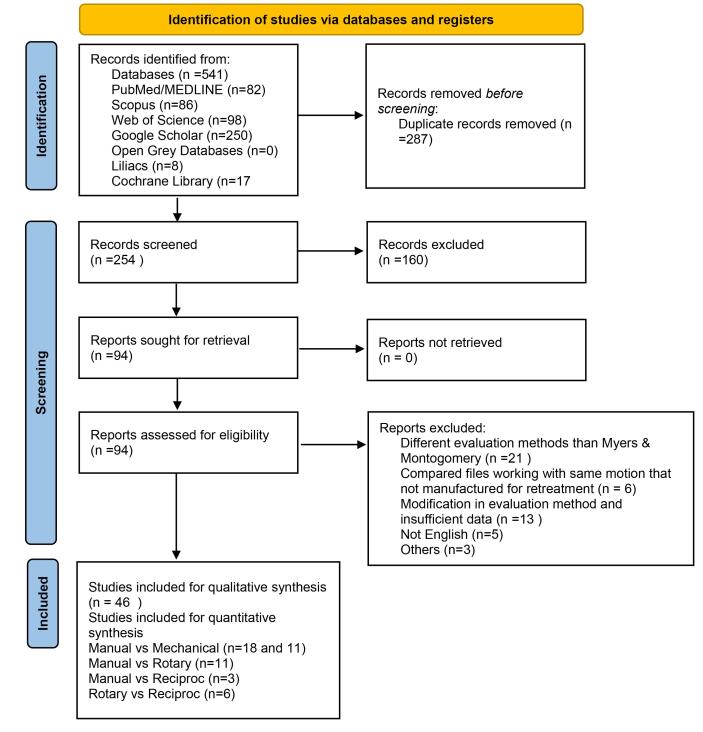
PRISMA flow diagram

 The database searches provided 541 results. Of these, 250 were in Google Scholar, 82 in Pubmed, 86 in Scopus, 98 in Web of Science, 8 in Lilacs, and 17 in Cochrane Library. The manual search of the reference lists of the included studies and the search for grey literature provided no additional studies. Following duplicate removal, 254 items remained. After data screening, ninety-four articles were selected for full-text reading based on titles and abstracts. Forty-eight articles were excluded because they did not meet the inclusion criteria.^46-93^ One of the main reasons for exclusion was that extrusion was evaluated with different methods instead of weighing debris, such as visual evaluation^51-53,74,81,87,89,90,93^ or measuring the volume of debris.^49,54,55^ Eleven articles were excluded because data regarding incubation conditions after NSRCRT procedures were missing.^47,65,67-71,73,79,80,83^ Supplementary Table 2 shows the excluded articles and the reasons for exclusion. After the full-text reading, 46 articles were found suitable for the present systematic review (Figure 1).^4,7-12,23-32,34-41,94-114^ The key characteristics of the included studies are shown in Supplementary Table 3. Single-rooted teeth were used in more than 80% of the studies.^7,9-12,23-32,36-38,40,41,94-105,108,110-114^ Only seven studies preferred to use molar teeth^4,8,34,35,39,106,107^ and one study did not provide information regarding the tooth type used.^109^ In more than 60% of the studies, researchers preferred to use straight roots/root canals (curvature < 10°)^4,7,9-12,24,25,27-30,32,36-38,40,41,94-98,101-105,112,114^ and in more than 90% of the studies, WL was determined 1 mm short of the major apical foramen.^4,7-12,23-32,34,35,37-41,95-98,100-110,112-114^ Cold lateral condensation was used as the obturation technique in more than half of the included studies.^7,9,11,12,23-25,27-30,34,38,41,96-98,99-103,107,112-114^ Resin-based sealers were the most selected sealer type in the included studies.^4,7,8,10-12,23-32,34-41,94-96,100,102-105,108,110,112,114^

 Periodontal ligament simulation was performed in two studies with thin silicone materials.^32,39^ In most of the studies, NSRCRT was performed without solvents.^4,8,23-25,29-32,34-41,94,95,98-102,105,106,109,111,114^ Eucalyptol was the most commonly used solvent among the studies.^7,26-28,107,112^ The most preferred solution during NSRCRT was distilled water^4,7,8,10-12,23,25-30,32,34,36,38,40,41,94-97,99-101,103,105-107,109-113^ followed by sodium hypochlorite with different concentrations between 1% and 5.25%.^9,31,35,37,39,98,102,114^

 Different incubation conditions were reported for debris drying ranging from 5 h to 4 weeks and 37 °C to 140 °C (Supplementary Table 3). In more than half of the studies, the diameter of the last instrument used at the WL was larger than the diameter of the master apical file used before obturation.^4,7,8,11,12,23,25,26,28-30,32,35,37-39,41,94,96,99,101,106,11,112^ Seven studies compared the effect of variables applied during the obturation phase,^11,12,39-41,111,112^ while 41 studies compared the effect of variables applied during the retreatment phase.^4,7-11,23-32,34-38,40,94-110,113,114^ Detailed results regarding the compared parameters in the included studies were given in the following paragraphs. The methodological risk of bias in the included studies is presented in Table 1.

**Table 1:** Risk of bias evaluation

**Reference**	**Author-Year**	**Sample size calculation**	**Apical diameter check during sample selection**	**Standardization of samples based on root canal shape**	**Quality of root canal obturation**	**Randomization**	**Manufacturer’s instructions during RT**	**Single and/or experienced operator during RT**	**Blinding of the operator during RT**	**Standard total volume of irrigants in groups during RT**	**Confirmation of Gutta-percha removal during RT, WL: working length; FC: file cleanliness; RCW: root canal wall cleanliness, RG: radiograph (at least with 1 parameter)**	**Total**	**Result**
^103^	Huang et al, 2007	0	1	0	1	1	1	1	1	1	1	8	L
^97^	Arora et al, 2012	0	0	0	1	1	0	1	1	1	1	6	M
^27^	Kuştarcı et al, 2012	0	0	0	1	1	1	1	1	1	1	7	M
^107^	de Morais Vitoriano et al, 2013	0	0	0	1	1	1	0	0	0	1	4	H
^98^	Arslan et al, 2014	0	1	0	1	1	0	0	1	1	1	6	M
^37^	Silva et al, 2014	0	1	0	1	1	1	1	1	1	1	8	L
^28^	Topçuoğlu et al, 2014	0	1	0	1	1	1	1	1	1	1	8	L
^12^	Çanakçı et al,2015	0	0	0	1	1	1	1	0	1	1	6	M
^38^	Dinçer et al, 2015	0	1	0	0	1	0	0	0	1	1	4	H
^11^	Türker et al, 2015	0	1	1	1	1	1	1	1	1	1	9	L
^29^	Altunbaş et al, 2016	0	1	0	1	1	1	0	1	0	1	6	M
^100^	Cakici et al, 2016	0	1	1	1	1	0	1	1	1	1	8	L
^23^	Çanakçı et al, 2016	0	0	0	0	1	1	1	0	1	1	5	M
^24^	Çiçek et al, 2016	0	0	0	0	1	0	0	1	0	1	3	H
^101^	Gkampesi et al, 2016	0	1	0	1	1	1	1	1	1	1	8	L
^9^	Kasam et al, 2016	1	0	0	0	1	0	0	0	0	1	3	H
^110^	Pawar et al, 2016	0	0	0	0	1	0	0	0	0	1	2	H
^25^	Uzunoglu & Türker 2016	0	1	1	1	1	1	1	1	0	1	8	L
^7^	Alfenas et al, 2017	0	0	0	1	1	1	1	1	1	1	7	M
^8^	Kaşıkçı Bilgi et al, 2017	1	1	0	1	1	1	1	1	1	1	9	L
^106^	Liu et al, 2017	0	1	0	1	1	1	1	1	1	1	8	L
^35^	Nevares et al, 2017	1	0	0	1	1	0	0	1	1	1	6	M
^113^	Vikram 2017	0	0	0	0	0	0	0	1	0	0	1	H
^32^	Yılmaz & Özyürek 2017	0	0	0	0	1	1	1	1	1	0	5	M
^36^	Azim et al, 2018	0	0	1	1	1	0	1	0	1	1	6	M
^34^	Delai et al, 2018	0	1	1	1	1	0	1	1	0	0	6	M
^104^	Jena et al, 2018	1	0	0	1	1	1	0	1	0	1	6	M
^114^	Pesic et al, 2018	0	0	0	1	1	1	0	1	0	1	5	M
^10^	Shivanna et al, 2018	0	0	1	0	1	0	0	1	0	1	4	H
^112^	Topçuoğlu et al, 2018	1	0	1	1	1	0	1	1	1	1	8	L
^99^	Balseca et al, 2019	1	0	0	1	1	0	0	1	1	0	5	M
^41^	Çanakçı et al, 2019	0	0	0	1	1	1	1	0	1	1	6	M
^96^	Sarıçam et al, 2019	1	0	0	0	0	1	1	0	1	0	4	H
^108^	Aldajani&Mathew 2020	0	1	1	0	1	0	1	1	0	1	6	M
^40^	Kamil &Al-Sabawi 2020	0	1	0	0	1	1	0	1	1	1	6	M
^26^	Li et al, 2020	0	0	0	1	1	0	0	0	0	1	3	H
^109^	Mircheska et al, 2020	0	0	0	0	0	1	0	0	0	0	1	H
^39^	Romeiro et al, 2020	1	0	0	1	1	0	1	1	1	1	7	M
^30^	Topçuoğlu et al,2020	1	0	1	1	1	0	1	1	1	1	8	L
^94^	Aktemur Türker&Kaşıkçı 2021	0	1	0	1	1	1	0	1	1	1	7	M
^95^	AlOmari et al, 2021	1	1	0	1	1	0	1	0	1	1	7	M
^31^	Dadalti et al, 2021	0	0	0	1	1	1	1	1	0	0	5	M
^111^	Pirani et al, 2021	1	0	0	1	1	1	1	1	1	1	8	L
^4^	Serefoğlu et al, 2021	1	1	0	1	1	1	1	1	1	1	9	L
^102^	Hassan et al, 2022	1	1	0	1	1	1	0	1	0	1	7	M
^105^	Karova et al, 2022	0	0	1	1	1	1	0	0	0	1	5	M

Abbreviations: RT: Retreatment, M: Medium, L: Low, H: High.

###  Obturation phase

 The effect of root canal sealers on AED was investigated in three studies.^12,39,112^ In two studies^39,112^ calcium-silicate-based sealers (CSB) were compared with resin-based sealers (RBS), and in one study, zinc oxide‒eugenol-based sealer (ZOEBS) was compared with RBS.^12^ According to these studies, there was no significant difference between the tested sealer groups regarding AED.^12,39,112^ While moderate evidence was obtained for the comparison of RBS and CSBS (two studies with a medium risk of bias),^39,112^ limited evidence was found for the comparison of RBS and ZOEBS (one study with a medium risk of bias).^12^

 The effect of root canal obturation techniques on AED was evaluated in 5 studies.^11,40,41,111,112^ Two studies with a low risk of bias compared the effect of cold lateral condensation and the single-cone techniques.^11,112^ Türker et al^11^ reported no significant difference between the techniques, while Topçuoğlu et al.^112^ reported that single-cone use decreased the amount of AED. Therefore, the evidence was conflicting in this regard. The effect of warm obturation techniques on AED was similar to cold obturation techniques in two studies (one with a low risk of bias^111^ and one with a medium risk of bias^40^). On the other hand, one study with a low risk of bias^112^ and one with a medium risk of bias^41^ reported that warm obturation techniques increased the amount of AED compared to cold obturation techniques. Therefore, the evidence was also conflicting in this regard.

###  Retreatment phase

####  The use of solvents during retreatment

 Six studies evaluated the effect of solvent use on AED.^7,10,11,26,107,108^ Different results were reported regarding this step. Two studies with a high risk of bias^26,107^ and one study with a medium risk of bias^7^ reported that solvent use did not affect the amount of AED, while one study with a low risk of bias^11^ and one with a high risk of bias^10^ reported that solvent use decreased the amount of AED. On the other hand, one study with a medium risk of bias^108^ reported that solvent use increased the amount of AED. Therefore, the evidence regarding the effect of solvents on the amount of AED was conflicting.

###  The use of a retreatment system

 Manufacturers have produced specific file systems for NSRCRT procedures, such as the ProTaper Universal retreatment system (PTUR, Dentsply Maillefer, Ballaigues, Switzerland), D-RaCe retreatment system (FKG Dentaire, La Chaux-de-Fonds, Switzerland), Mtwo retreatment system (VDW, Munich, Germany), R-Endo retreatment system (Micro-Mega, Besancon, France), EdgeFile retreatment system (EdgeEndo, Albuquerque, NM, USA), Endostar retreatment system (Poldent Co. Ltd., Warsaw, Poland), and XP finisher R file (FKG Dentaire SA, La Chaux-de-Fonds, Switzerland). In eleven studies, none of these retreatment systems were used.^26,29,32,35,36,39,94,95,106,108,111^ In these studies, reciprocating files such as Reciproc (VDW, Munich, Germany),^29,32,35,39,94,95^ Reciproc Blue (VDW, Munich, Germany),^39^ and WaveOne Gold (Dentsply Sirona, Ballaigues, Switzerland)^36^ or rotary files such as ProTaper Gold (Dentsply Maillefer, Ballaigues, Switzerland)^108^ and ProTaper Next (Dentsply Maillefer, Ballaigues, Switzerland)^32,35^ were used. Also, recently manufactured files such as XP Shaper (FKG, La Chaux de Fonds, Switzerland),^36,95^ Hyflex EDM (Coltene/Whaledent GmbH + Co. KG Germany),^36,108,111^ and Twisted File (SybronEndo, Orange, CA, USA)^29,32,106^ were tested. Among the included studies, PTUR was the most selected retreatment system.^4,8-12,23,24,28,30,31,34,37,38,41,96-98,99-101,103,104,107,109,110,112-114^ Comparative findings on the effect of PTUR and other retreatment systems on the amount of AED are as follows:

 PTUR and Mtwo retreatment systems were compared in seven studies.^10,23,24,31,38,100,101^ One study with a high risk of bias reported that PTUR increased the amount of AED compared to the Mtwo retreatment system,^10^ while one study with a medium risk of bias reported the opposite.^31^ Two other studies with a low risk of bias,^100,101^ one with a medium risk of bias,^23^ and two with a high risk of bias^24,38^ reported that both systems resulted in a similar amount of AED. Therefore, moderate evidence was found in favor of similar effects of these retreatment systems.

 Six studies compared the effect of PTUR and D-RaCe retreatment systems.^23,28,30,34,98,114^ Only one study with a medium risk of bias reported that PTUR increased the amount of AED^23^ compared to the D-RaCe retreatment system. The two remaining studies with a low risk of bias^28,30^ and three with a medium risk of bias^34,98,114^ reported that both systems resulted in a similar amount of AED. Therefore, strong evidence was found in favor of similar effects of these retreatment systems.

 The effects of PTUR and R-Endo retreatment systems were compared in six studies.^4,8,23,28,98,101^ Only one study with a medium risk of bias reported that PTUR increased the amount of AED^23^ compared to the R-Endo retreatment system. The four remaining studies with a low risk of bias^4,8,28,101^ and one with a medium risk of bias^98^ reported that both systems resulted in a similar amount of AED. Therefore, strong evidence was found in favor of similar effects of these retreatment systems.

 The effects of PTUR and Endostar retreatment systems were compared in one study with a medium risk of bias.^104^ There was no significant difference between the systems regarding the amount of AED. Therefore, limited evidence was found for the similar effects of these retreatment systems.

 The remaining comparisons of other retreatment systems are presented in Table 2.

**Table 2:** Level of evidence results of different retreatment systems comparisons

**Study**	**RS Comparison**	**Risk of bias**	**Level of evidence**
Arslan et al, 2014^98^	D-RaCe = R-Endo	M	D-RaCe RS vs R-Endo RS: Modarate evidence for D-RaCe RS = R-Endo RS
Topçuoğlu et al, 2014^28^	D-RaCe = R-Endo	L	
Çanakçı et al, 2016^23^	Mtwo > D-Race = R-Endo	M	D-RaCe RS vs EdgeFile RS: Modarate evidence for D-RaCe RS = EdgeFile RS
Gkampesi et al, 2016^101^	Mtwo = R-Endo	L	
Uzunoglu & Türker, 2016^25^	D-RaCe > EdgeFileXR	L	Mtwo RS vs D-RaCe RS: Conflicting evidence for Mtwo RS = D-RaCe RS
Kamil & Al-Sabawi, 2020^40^	D-RaCe > EdgeFileXR	M	
Karova et al, 2022^105^	Mtwo = D-RaCe	M	Mtwo RS vs R-Endo RS: Conflicting evidence for Mtwo RS = R-Endo RS

Abbreviations: RS: Retreatment System, M: Medium, L: Low. = : No significant difference, >: Significantly higher, amount of extruded debris sequenced from the most to the least

###  The use of additional file systems after instrumentation with retreatment systems 

 In 12 studies, retreatment was completed with only one of the retreatment systems mentioned above,^9,10,31,34,40,97,98,104,105,107,109,113^ while in 21 studies, retreatment was completed with the use of additional rotary or reciprocating file systems after instrumentation with one of the retreatment systems.^4,7,8,11,12,23,25,27,28,30,37,38,41,96,98,99,101-103,112,114^ However, only four studies evaluated whether the use of additional file systems had an impact on the amount of AED.^24,94,100,110^ Of these studies, one presented low,^100^ one presented medium,^94^ and two presented high risk of bias.^24,110^ Cakıcı et al^100^ and Türker et al^94^ reported that additional file use did not affect the amount of AED, while Çiçek et al^24^ and Pawar et al^110^ reported that additional file use increased the amount of AED. Therefore, conflicting evidence was found in this regard.

###  Manual instrumentation vs. engine-driven instrumentation 

 Twenty-three studies compared the effect of manual instrumentation with hand files and engine-driven instrumentation with rotary, reciprocating files, or ultrasonic tips.^4,7-10,27,31,34,38,96,98,101-103,106,107,109,113,114^ Of these studies, seven presented low^4,8,28,30,101,103,106^; nine presented medium,^7,27,29,31,34,97,98,102,114^ and seven presented high risk of bias.^9,10,38,96,107,109,113^ Four studies with a low risk of bias reported that manual instrumentation extruded more debris apically compared to engine-driven instrumentation.^8,28,30,106^ Furthermore, 78% of the studies reported that manual instrumentation extruded more debris apically compared to engine-driven instrumentation.^8-10,27-30,34,38,96,97,101-103,106,109,113,114^ Therefore, strong evidence indicated the increased debris extrusion potential of manual instrumentation compared to engine-driven instrumentation.

###  Manual instrumentation vs. rotary instrumentation 

 Since there was at least one rotary group in the 23 studies above, the same studies were also evaluated in this section.^4,7-10,27-31,34,38,96-98,101-103,106,107,109,113,114^ Overall, 15 studies reported manual instrumentation during NSRCRT extruded more debris apically compared to rotary instrumentation,^9,10,27-30,96,97,101-103,106,109,113,114^ and three of them presented a low risk of bias.^28,30,106^ Eight studies reported no difference between the amount of AED with both instrumentation types.^4,7,8,31,34,38,98,107^ Therefore, there is moderate evidence that rotary instrumentation has less residual extrusion potential than manual instrumentation.

###  Manual instrumentation vs. reciprocating instrumentation 

 Seven studies compared the effect of manual instrumentation and reciprocating instrumentation.^4,8,29-31,34,38^ Two studies with low,^8,30^ two with medium,^29,34^ and one with high risk of bias^38^ reported that hand files extruded more debris apically compared to reciprocating files. Two studies (one with low^4^ and one with medium risk of bias^31^) reported no significant difference between the amount of AED with both instrumentation types. Therefore, there is moderate evidence that reciprocating instrumentation has less residual extrusion potential than manual instrumentation.

###  Rotary instrumentation vs. reciprocating instrumentation 

 Fifteen studies^4,8,23,25,29-32,34-38,95,99^ compared the effects of rotary files and reciprocating files. Five studies (two with low,^25,37^ two with medium,^31,34^ and one with high risk of bias^38^) reported that rotary files extruded more debris apically compared to reciprocating files, while two studies with medium risk of bias reported the opposite.^23,32^ Eight studies (three with low^4,8,30^ and five with medium risk of bias^29,35,36,95,99^) reported no significant difference between the amount of AED with both instrumentation types. Therefore, conflicting evidence was found in this regard.

###  Meta-analysis

 Quantitative analysis could be performed for the effect of instrumentation types on the amount of AED as the studies provided adequate data to be combined.

###  Manual instrumentation vs. engine-driven instrumentation 

**Figure 2:**
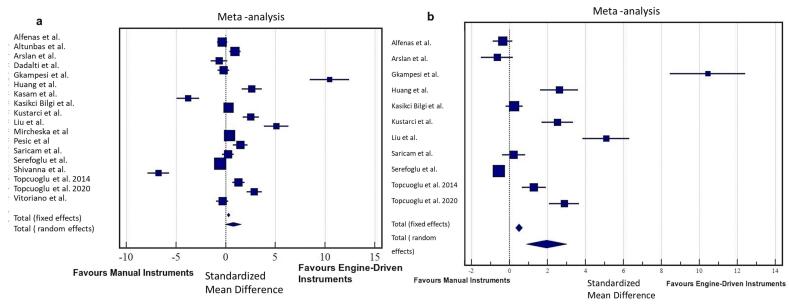
a. Forest plot of standardized mean difference (SMD) with 95% confidence interval (CI) of the apically extruded debris weight using manual instruments vs. engine-driven instruments. b. Forest plot of SMD with 95% CI of the apically extruded debris weight using manual instruments vs. engine-driven instruments, excluding studies that used different amounts of root canal irrigants

 Eighteen studies were included in this analysis.^4,7-10,27-31,96,98,101,103,106,107,109,114^ A random-effects model was used (I^2^ = 96.90%, *P* < 0.0001), which revealed no significant difference in the amount of AED between manual and engine-driven instrumentation techniques [SMD: 0.74, 95% confidence interval (CI): -0.086‒1.573, *P* > 0.05] (Figure 2a).

 A previous study reported that irrigation itself may play an important role in the amount of AED.^115^ Considering this, a meta-analysis was also performed with studies that specifically used the same irrigant volume in groups during NSRCRT procedures.^4,7,8,27,28,30,96,98,101,103,106^ Interestingly, this time, a random-effects model (I^2^ = 96.71%, *P* < 0.0001) revealed a significant difference in the amount of AED between manual and engine-driven instrumentation techniques (SMD: 1.95, 95% CI: 0.888‒3.003, *P* < 0.001) (Figure 2b). Considering this result, quantitative analyses of the following subgroups were performed with studies reporting the use of the same irrigant volume in groups during the NSRCRT.

###  Manual instrumentation vs. rotary instrumentation

**Figure 3:**
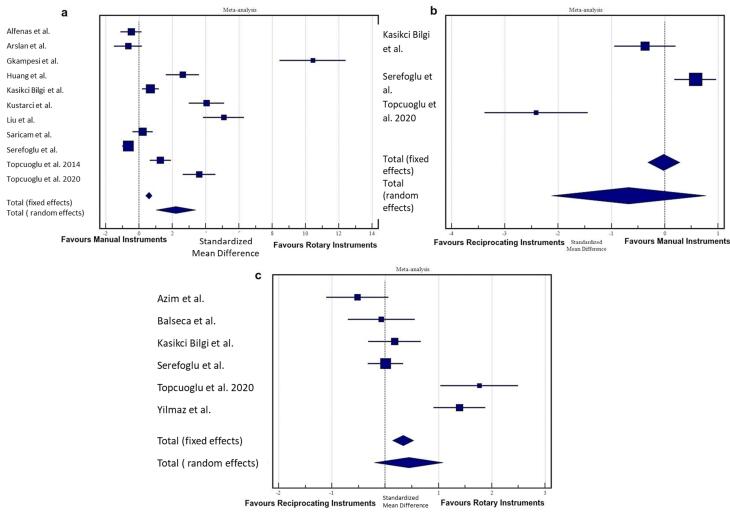
a. Forest plot of standardized mean difference (SMD) with 95% confidence interval (CI) of the apically extruded debris using manual instruments vs. rotary instruments. b. Forest plot of SMD with 95% confidence interval (CI) of the apically extruded debris using reciprocating instruments vs. manual instruments. c. Forest plot of SMD with 95% confidence interval (CI) of the apically extruded debris using reciprocating instruments vs. rotary instruments

 Eleven studies were included in this subgroup analysis.^4,7,8,27,28,30,96,98,101,103,106^ Significant heterogeneity was found (I^2^ = 96.95%, *P* < 0.0001). A random-effects model revealed that manual instrumentation resulted in a higher amount of AED than rotary instrumentation during NSRCRT (SMD: 2.21, 95% CI: 1.021‒3.395, *P* < 0.001) (Figure 3a).

###  Reciprocating instrumentation vs. manual instrumentation 

 Three studies were included in this subgroup analysis.^4,8,30^ Significant heterogeneity was found (I^2^ = 94.46%, *P* < 0.0001). A random-effects model revealed no significant difference in the amount of AED between hand and reciprocating instruments during NSRCRT (SMD: -0.678, 95% CI: -2.126‒0.769, *P* = 0.356) (Figure 3b).

###  Reciprocating instrumentation vs. rotary instrumentation 

 Six studies were included in this subgroup analysis.^4,8,30,32,36,99^ A random-effects model (I^2^ = 89.79%, *P* < 0.0001) revealed no significant difference in the amount of AED between the rotary and reciprocating instruments during NSRCRT (SMD: 0.44, 95% CI: -0.205‒1.087, *P* = 0.181) (Figure 3c).

## Discussion

 This systematic review and meta-analysis were performed to reveal the effects of various variables in the NSRCRT procedure on AED. The treatment variables evaluated in the included studies can be listed as obturation technique and sealer type during the filling of root canals and solvent use, retreatment system, and motion kinematics during the removal of filling materials. According to this review, most of the studies examined the effects of variables in the retreatment phase rather than in the obturation phase. Therefore, the evidence level for treatment variables in the obturation phase was generally limited or conflicting due to the small number of studies. In terms of variables in the retreatment phase, strong evidence showed that manual techniques cause more AED than engine-driven techniques during the removal of filling materials.

 Despite the limited number of studies, the present findings show that treatment variables in the obturation phase do not have a prominent effect on debris extrusion. Resin-based sealers were compared with calcium silicate- and zinc-oxide eugenol-based sealers in the studies, and there were no differences between sealers regarding AED because of their similar physical properties. It has been reported that resin- and calcium silicate-based sealers revealed similar dentinal tubule penetration and removability patterns.^116,117^ Conflicting results were obtained from the studies that compared the effect of warm (continuous wave, warm vertical compaction, and carrier-based obturation) and cold obturation techniques (single cone and cold lateral condensation) on AED.^40,41,111,112^ This could be because of the different application steps of each obturation technique. Some of the included studies reported that the warm vertical compaction technique increased the amount of AED compared to cold lateral condensation^41,112^ and single cone techniques^112^ because the warm vertical compaction technique provides more homogenous dense root canal filling^118^ with a lower percentage of voids^119^ and greater mass of gutta-percha^118^ than cold obturation techniques. However, the available data do not provide a high level of evidence nor allow quantitative analysis; therefore, further studies are required to clarify the effect of obturation techniques on AED.

 There were some variables in the retreatment phase of the included studies, including the type of irrigant used. Distilled water was the irrigant of choice in most of the studies included, while NaOCl was used in a few studies. The reason for using NaOCl in these studies is probably to simulate clinical conditions as much as possible.^37^ On the other hand, it has been reported that NaOCl crystallizes after evaporation, increasing the amount of AED.^120^ For these reasons, it may be more reasonable to use distilled water during NSRCRT in ex vivo studies measuring the amount of AED. However, the literature on the effect of different irrigants on the amount of AED is lacking, so it is difficult to reach a specific conclusion. The quantitative analysis in the present study revealed that not using the same volume of irrigant in each group can be a confounding factor when comparing the amount of AED. When all eligible studies were combined in the meta-analysis,^4,7-10,27-31,96,98,101,103,106,107,109,114^ no significant difference was found in the amount of AED between the manual and engine-driven instrumentation techniques. On the other hand, when the studies using the same irrigant volume in the groups were combined,^4,7,8,27,28,30,96,98,101,103,106^ it was found that the manual instrumentation technique extruded significantly more debris than the engine-driven instrumentation technique. Therefore, using irrigants at different volumes in groups could be the reason for this finding. Interestingly, Vande Visse and Brilliant^115^ reported that irrigation itself plays an important role in collectible debris. Collectible debris was observed when an irrigant was used; however, there was no debris extrusion when an irrigant was not used. The effect of volume and type of irrigants used during NSRCRT on AED must be clarified with further studies.

 The findings in favor of rotary instrumentation compared to manual instrumentation can be explained by the fact that rotary instruments move debris coronally instead of compacting it apically.^27^ Furthermore, early flaring of the coronal third may improve instrument control during the reshaping of the apical third. This may also prevent the friction of the instrument and pressure on it in the root canal, which could increase AED. On the other hand, the quantitative analysis revealed no significant difference in the amount of AED between manual and reciprocating instrumentation, which might be related to the low number of studies combined for this analysis,^4,8,30^ as the use of reciprocating instruments was found to extrude less debris compared to hand instruments in two studies.^8,30^ Reciprocating systems are single-file systems, so the number of instruments used in this technique is low compared to manual instrumentation techniques, and coronal enlargement is performed in the early stages of root canal preparation during reciprocating instrumentation, similar to rotary instrumentation, which might have contributed to less debris extrusion with reciprocating instrumentation in these studies.^8,30^

 The quantitative analysis for the effect of rotary and reciprocating instruments on AED during NSRCRT revealed no significant difference between them.^4,8,30,32,36,99^ A recent systematic review concluded that the use of reciprocating instruments increased the amount of AED compared to rotary instruments.^121^ The different results between the studies may be due to differences in the research question. The current review included the studies that performed retreatment, while the previous review included the studies that performed initial root canal treatment.^121^ Furthermore, the previous review evaluated only single-file systems,^121^ while the current review was conducted without restriction on the number of files. It is also important to mention that most of the studies included in the current review used rotary file systems specifically manufactured for NSRCRT procedures. However, reciprocating files were manufactured for initial root canal treatment instead of retreatment.

 In some of the included studies, special file systems manufactured only for NSRCRT procedures were used. Among these systems, the most frequently used system was PTUR, followed by D-RaCe, Mtwo, and R-Endo retreatment systems, respectively. Strong evidence was found regarding the similar extrusion potential of PTUR with D-RaCe and R-Endo systems. In contrast, moderate evidence was found regarding similar extrusion potential of PTUR and Mtwo retreatment systems. Therefore, it can be concluded that retreatment systems have a similar effect regarding the apical extrusion of debris.

 Another treatment variable in the NSRCRT phase was the use of solvents during the removal of filling materials. Based on the present review, inconsistent findings were reported regarding the effect of solvent use on AED.^7,10,11,26,107,108^ Solvents can make the removal of filling materials easier and quicker.^11,26^ However, it may negatively affect the cleanliness of root canal walls^122,123^ and present cytotoxicity.^124^ Therefore, the use of solvents during NSRCRT is controversial.

 Many studies reported the importance of using a larger file during the NSRCRT procedure than the master file used in the initial treatment to improve the cleanliness of root canals.^11,125^ Twenty-four of the included studies ensured this procedure in all groups^4,7,8,11,12,23,25,26,28-30,32,35,37-39,41,94,96,99,101,106,11,112^; however, in 11 studies retreatment procedures were completed with either smaller files or with similar files compared to the master file used during initial treatment.^9,10,27,31,34,36,40,98,104,105,114^ The standardization in this step can help obtain results that are in line with the clinical goals, such as the complete removal of filling materials from the root canal system.

 The amount of AED can be evaluated with different methods, such as measuring debris weight, using three-dimensional imaging, and evaluating neuropeptide release, bacteria extrusion, or irrigant extrusion.^20^ According to a recent critical review, many studies adopted the methodology of Myers and Montgomery^126^ to evaluate the amount of AED.^20^ In this method, debris is collected in an empty pre-weighed tube and re-weighed after an evaporation step to obtain dry debris.^126^ This method offers advantages such as practicality, simplicity, reproducibility, and possible comparison between treatment variables.^20^ Considering its advantages and popularity, studies that followed a protocol similar to the Myers and Montgomery method^126^ were included in the present review. Including studies with a similar method allowed us to obtain more comparable results. On the other hand, it is important to mention that notable differences were observed in eliminating irrigants from collector tubes between the studies. Different incubation conditions ranging from 5 h to 4 weeks and 37 °C to 140 °C were reported to obtain dry debris in the included studies. No ideal conditions have been reported for this step, and the effect of different durations or temperatures on the debris weight has not been investigated in the literature.

 In this review, many parameters were considered when evaluating the risk of bias in the included studies. Sample size calculation, which was reported as a factor directly affecting the study’s results, was one of the parameters.^127^ Internal and external validity of the study is undermined with very small samples, while very large samples may play a role in statistical significance.^127^ In more than 70% of the included studies, sample size calculation was not performed before the experiments, which increased the risk of bias. Standardization of samples in terms of apical diameter and root canal shape may significantly impact the reliability of the results.^4,30,128^ Root canal shape could affect the volume of filling materials in the initial treatment, and apical diameter could affect the extrusion potential. Less than half of the included studies (41%) reported that initial apical diameter was standardized during sample selection, while only 22% of the included studies reported that the root canal shape of samples was standardized. Furthermore, 26% of the included studies did not check root canal obturation quality. Obturation quality might play a role in the amount of AED, and assigning poorly obturated samples to one group may increase the risk of bias. On the other hand, it is important to mention that in clinical situations, NSRCRT is generally performed in root canals that are poorly obturated. However, for the standardization of ex vivo studies, obturation quality should be checked to ensure adequate filling of samples. It is known that randomization prevents the selection bias and produces the comparable groups.^129^ Randomisation was performed in 93% of the studies, decreasing the risk of bias. Performing removal procedures by a single/experienced operator following the manufacturer’s instructions may also increase the quality of a study. It was reported that operator variations could affect the results of a study.^130^ Therefore, conducting all NSRCRT procedures by a single/experienced operator according to the manufacturer’s instructions would be beneficial for the reliability of the study, and this was performed by less than half of the included studies (n = 18). Blinding of the operator is another parameter that can minimize bias and increase validity;^131^ around 72% of the included studies provided this parameter. Studies should also consider standardizing NSRCRT completion steps such as reaching WL, taking radiographs to confirm complete removal, or checking files for cleanliness. At least one of these steps was reported in 85% of the included studies and reduced the risk of bias. Standardization of irrigant volume in groups during the NSRCRT procedure was another parameter taken into account when evaluating the risk of bias, and the effect of this parameter on the results has been discussed in detail in the previous paragraphs. In 63% of the included studies, the standard volume of irrigant was used in groups during NSRCT procedures. All these variables may affect the results and prevent reproducibility. It is recommended that future studies consider all these parameters when conducting experiments to obtain more reproducible and reliable data.

## Conclusion

 The current study systematically reviewed the effects of treatment variables on AED following root canal retreatment procedures. It can be concluded that engine-driven instruments, especially rotary instruments, decreased the amount of AED compared to manual instruments. Therefore, the use of rotary instruments can be recommended during the removal of filling materials to control the amount of AED. Further studies with a low risk of bias are needed to obtain a high level of evidence for the effect of other variables such as type of sealer, obturation technique, use of solvent, and use of reciprocating instruments.

## Acknowledgments

 The authors are grateful to the researchers who shared their missing data.

## Competing Interests

 The authors declare that they have no conflict of interest.

## Ethical Approval

 Not applicable.

## Funding

 None.

## Supplementary Files

Supplementary Table 1. Examples of the search strategy of databases

Supplementary Table 2. The excluded articles and the reasons for exclusion

Supplementary Table 3. Extracted variables from the included articles
