# Supplementary material for: Effect of treatment variables on apical extrusion of debris during root canal retreatment: A systematic review and meta-analysis of laboratory studies
Source: J Dent Res Dent Clin Dent Prospects. 2024 Mar 29;18(1):1–16. doi: 10.34172/joddd.40501 (PMC11179139; doi:10.34172/joddd.40501)
Supplement: Supplementary file 1 — Supplementary Table 1. Examples of the search strategy of databases [file joddd-18-1-s001.pdf]

Uzunoğlu-Özyürek et al, J Dent Res Dent Clin Dent Prospects, 2024, 18(1), 1-S1

doi: 10.34172/joddd.40501

https://joddd.tbzmed.ac.ir

Supplementary Table 1: Search strategies for databases

Example of the search strategy (Web Of Science)

No	Search Strategy	Results
1	TS=(extrusion or extruded)	119563
2	TS=(debris or gutta-percha or gutta percha or sealer or sealant or filling material)	373464
3	TS=(retreatment)	18076
4	TS=(endodontic or root canal)	35955
5	#4 AND #3 AND #2 AND #1	98

Example of the search strategy (Pubmed)

No	Search Strategy	Results
1	extrusion or extruded	34262
2	debris or gutta-percha or gutta percha or sealer or sealant or filling material	70874
3	retreatment	25683
4	endodontic or root canal	63100
5	(((extrusion or extruded) AND (debris or gutta-percha or gutta percha or sealer or sealant or filling material)) AND (retreatment)) AND (endodontic or root canal)	82
