# Supplementary material for: Effect of treatment variables on apical extrusion of debris during root canal retreatment: A systematic review and meta-analysis of laboratory studies
Source: J Dent Res Dent Clin Dent Prospects. 2024 Mar 29;18(1):1–16. doi: 10.34172/joddd.40501 (PMC11179139; doi:10.34172/joddd.40501)
Supplement: Supplementary file 2 — Supplementary Table 2. The excluded articles and the reasons for exclusion [file joddd-18-1-s002.pdf]

Uzunoğlu-Özyürek et al, J Dent Res Dent Clin Dent Prospects, 2024, 18(1), 1-S2

doi: 10.34172/joddd.40501

https://joddd.tbzmed.ac.ir

Supplementary Table 2: Excluded articles and reasons for exclusion

**Reference Number**	**Excluded Articles**	**Reasons for Exclusion**	**Compared paramateres**
48	Aun&Santos 1989	Visually observed	Manual/Engine Driven
71	Ladley et al.1991	Storage condition after retreatment procedure not available, Used aluminum crown instead of eppendorf tubes	Manual/Engine Driven
68	Imura et al. 1996	Storage condition after retreatment procedure not available, Used aluminum foil instead of eppendorf tubes	Manual/Engine Driven
65	Hülsmann&Stotz 1997	Storage condition after retreatment procedure not available, Used stainless steel crown instead of eppendorf tubes	Manual/Engine Driven, Solvent/Nosolvent
60	Frajlich et al. 1998	Visually observed	Obturation techniques
53	Bramante&Betti 2000	Visually observed and scored, Compared only rotating file systems	Same rotating with diffrent rpm
67	Imura et al. 2000	Storage condition after retreatment procedure not available, Used paper filters instead of eppendorf tubes	Manual/Engine Driven
51	Betti&Bramante 2001	Visually observed and scored	Manual/Engine Driven
64	Hülsmann&Bluhm 2004	Visually observed	Manual/Engine Driven
61	Ghoddusi et al. 2005	Not English	Manual/Engine Driven, Solvent/Nosolvent
81	Schirrmeister et al.2006	Visually observed and scored	Manual/Engine Driven

79	Saad et al. 2007	Storage condition after retreatment procedure not available, Used aluminum crown instead of eppendorf tubes	Manual/Engine Driven
87	Somma et al. 2008	Visually observed and scored	Manual/Engine Driven
89	Unal et al. 2009	Visually observed and scored	Manual/Engine Driven
88	Uezu et al. 2010	Compared only rotating file systems	Different rotating systems
46	Al-Haddad et al. 2011	Main filling Material: RealSeal	Manual/Engine Driven, Different RS
77	Pirovano et al. 2011	Not English	Different RS
75	Mollo et al. 2012	Visually observed	Manual/Engine Driven, Different RS
47	Al-Sabawi 2013	Storage condition after retreatment procedure not available	Manual/Engine Driven
56	Deonizio et al. 2013	Used paper filters instead of eppendorf tubes	Manual/Engine Driven
73	Lu et al. 2013	Storage condition after retreatment procedure not available	Manual/Engine Driven
78	Rehman et al. 2013	Visually observed	Different Solvents
82	Shivanand et al. 2013	Visually observed	Manual/Engine Driven
93	Yadav et al. 2013	Visually observed and scored	Manual/Engine Driven, Different RS
55	Chandrasekar et al. 2014	Extruded material measured as volume via spiral CT scan, Compared only reciprocating file systems	Manual/Engine Driven
74	Maiti et al. 2014	Visually observed and scored	Manual/Engine Driven, Different RS
83	Sihivahanan et al. 2015	Storage condition after retreatment procedure not available	Manual/Engine Driven, Different RS, RBS vs ZOEBS
85	Silva et al. 2015	Visually observed	Rotary vs Reciprocation
49	Azevedo et al. 2016	Extruded material measured as volume via microct images	Manual/Engine Driven
62	Mora Gonzalez et al. 2016	Not English	Solvent/Nosolvent
84	Silva et al. 2016	Evaluated the cytotoxic effects of the debris apically extruded during root canal retreatment	Manual/Engine Driven

50	Baybora et al. 2017	Used immature samples	Manual/Engine Driven
69	Keskin et al. 2017	Storage condition after retreatment procedure not available, Used agar gel around eppendorf tubes	Solvent/Nosolvent
72	Li et al. 2017	Not English	Manual/Engine Driven
80	Saba et al. 2017	Storage condition after retreatment procedure not available	Different RS, Sealers (2 RBS)
58	Dixit et al. 2018	Compared only rotating file systems	Different rotating systems
66	Hussein&Bayoumi 2018	Compared only rotating file systems	Different rotating systems
70	Keskin&Sariyilmaz 2018	Storage condition after retreatment procedure not available, Used agar gel around eppendorf tubes	Rotary vs Reciprocation, Different RS
54	Canali et al. 2019	Extruded material measured as volume via microct images	Different reciprocating systems
63	Gupta et al. 2019	Compared only rotating file systems	Different rotating systems
59	DoğanayYıldız&Arslan 2019	Compared only reciprocating file systems	Different reciprocating systems
52	Boboc et al. 2020	Visually observed and scored	Manual/Engine Driven
86	Solda et al. 2020	Used paper filters instead of eppendorf tubes	Rotary vs Reciprocation
90	Vokuje et al. 2020	Visually observed and scored	Manual/Engine Driven
91	Xu et al. 2020	Resin blocks used instead of extracted teeth	Manual/Engine Driven
57	Dhaimy et al. 2021	Extruded material measured as area via software program	File vs additional file
92	Yilmaz&Tüfenkçi 2021	Compared only rotating file systems	Same system with diffrent rpm
76	Özata et al. 2022	Not English	Rotary vs Reciprocation
*Abbreviations: RS: Retreatment System, RBS: Resin-based sealer, ZOEBS: Zinc-oxide Eugenol-based sealer			
