# Supplementary material for: Effect of treatment variables on apical extrusion of debris during root canal retreatment: A systematic review and meta-analysis of laboratory studies
Source: J Dent Res Dent Clin Dent Prospects. 2024 Mar 29;18(1):1–16. doi: 10.34172/joddd.40501 (PMC11179139; doi:10.34172/joddd.40501)
Supplement: Supplementary file 3 — Supplementary Table 3. Extracted variables from the included articles [file joddd-18-1-s003.pdf]

Uzunoğlu-Özyürek et al, J Dent Res Dent Clin Dent Prospects, 2024, 18(1), 1-S3

doi: 10.34172/joddd.40501

https://joddd.tbzmed.ac.ir

Supplementary Table 3. Extracted variables from the included articles

Reference Number	Author	Year	Journal Name	Ethics Approval	Tooth Type	Root Canal curvature	Working Length	Final file used before obturation	Obturation technique	Obturation Materials	Storage conditions after obturation	Debris Collection	PDL Simulation	RT methods (final instrument used at WL)	solvent use during RT	Irrigation solutions used during RT	Patency after RT
**103**	Huang et al.	2007	Journal of Endodontics	NM	Max Incisors	CC<10°	1 mm short of AF	K files #30/.02	CLC	GP+RBS	37°C in a humidor for 1 month	Eppendorf Tubes	NM	Group I: PTUR + PTU F3<br>Group II: GGI-III+H files +PTU F3+solvent<br>Group III: GGI-III+H files +K-flex Files (35/.02)+ solvent	Y (chloroform)/ NO	DW	NM
**97**	Arora et al.	2012	Saudi Endodontic Journal	NM	Mand Premolar	CC<10°	1 mm short of AF	K files #30/.02	CLC	GP+ZOEBS	100% humidity at 37°C for 1 month	Eppendorf Tubes	NM	Group I: PTUR<br>Group II: K3 files<br>Group III: H files +solvent	Y (tetrachloroethylene)/NO	DW	NM
**27**	Kuştarcı et al.	2012	Journal of Dental Sciences	NM	Mand Premolar	CC<10°	1 mm short of AF	K files #30/.02	CLC	GP+RBS	100% humidity at 37°C for 2 weeks	Eppendorf Tubes	NM	Group I: K3 (30/.04)+ solvent<br>Group II: R-Endo RS+Rs(30/.04)+solvent<br>Group III: H files (30/.02)+ solvent	Y (eucalyptol)	DW	NM
**107**	de Morais Vitoriano et al.	2013	Revista Sul-Brasileira de Odontologia	Y	Mand Molars	CC<30°	1 mm short of AF	K3 files #30/.06	CLC	GP+ZOEBS	Humid conditions	Glass flasks	NM	Group I: K files (40/.02)<br>Group II: K files (40/.02)+ solvent<br>Group III:	Y (eucalyptol)/NO	DW	Y

														PTUR<br>Group IV: PTUR+solvent			
**98**	Arslan et al.	2014	Turkiye Klinikleri Journal of Dental Sciences	NM	Max Incisors	straight canals	1 mm short of AF	ProFile #30/.04	CLC	GP+CHBS	100% humidity at 37°C for 1 month	Eppendorf Tubes	NM	Group I: GGII-III+K Files (30/.02)<br>Group II: R-Endo RS<br>Group III: PTUR<br>Group IV: D-RaCe RS	NO	5% NaOCl	NM
**37**	Silva et al.	2014	Journal of Endodontics	Y	Mand Premolar	CC<10°	1 mm short of AF	ProTaper F3	continuous wave of condensation	GP+RBS	100% humidity at 37°C for 1 month	Plastic Tubes	NM	Group I: PTUR+ PTU F4<br>Group II: Reciproc R25+Reciproc R40<br>Group III: WaveOne Primary + WaveOne Large	NO	5.25% NaOCl +17% EDTA	NM
**28**	Topçuoğlu et al.	2014	Journal of Endodontics	NM	Mand Premolar	CC<5°	1 mm short of AF	K files #35/.02	CLC	GP+RBS	100% humidity at 37°C for 2 weeks	Eppendorf Tubes	NM	Group I: GGII-III+H files (40/.02)+ solvent<br>Group II: PTUR+PTU F4+solvent<br>Group III: D-RaCe RS + BR5(40/.04) + solvent<br>Group IV: R-Endo RS+ HERO642 (40/.02)+ solvent	Y (eucalyptol)	DW	NM
**12**	Çanakçı et al.	2015	Journal of Endodontics	NM	Mand Incisors	CC<10°	1 mm short of AF	Reciproc R25 (#25/.08)	CLC	GP+RBS or GP+ZOEBS	100% humidity at 37°C for 2 months	Tubes	NM	Group I: GGII-III+PTUR+ Reciproc R40(40/.06) +solvent	Y (Resosolv, Endosolv, Guttasolv)	DW	NM

**38**	Dinçer et al.	2015	International Endodontic Journal	NM	Mand Incisors	CC<10°	1 mm short of AF	Reciproc R25 (#25/.08)	CLC	GP+RBS	100% humidity at 37°C for 2 months	Glass vials	NM	Group I: PTUR+PTU F4<br>Group II: Mtwo RS+ Mtwofiles (40/.06)<br>Group III: Reciproc R25+ Reciproc R40(40/.06)<br>Group IV: GGI-III+H files (40/.02)	NO	DW	NM
**11**	Türker et al.	2015	Nigerian Journal of Clinical Practice	NM	Mand Premolar	CC<5°	1 mm short of AF	ProTaper F3	CLC or SC	GP+RBS	100% humidity at 37°C for 2 weeks	Eppendorf Tubes	NM	Group I: PTUR+PTU F4+solvent<br>Group II: PTUR+PTU F4	Y (chloroform)/ NO	DW	NM
**29**	Altunbaş et al.	2016	Journal of Istanbul University Faculty of Dentistry	NM	Mand Premolar	straight root canals	1 mm short of AF	ProTaper F3	CLC	GP+RBS	100% humidity at 37°C for 1 week	Eppendorf Tubes	NM	Group I: Reciproc R40 (40/.06)<br>Group II: Twisted Files (40/.04)<br>Group III: H files (40/.02)	NO	DW	NM
**100**	Cakici et al.	2016	The International Journal of Artificial Organs	NM	Mand Incisors	CC >5°	1 mm short of AF	ProTaper F2	CLC	GP+RBS	100% humidity at 37°C for 2 weeks	Eppendorf Tubes	NM	Group I: PTUR<br>Group II: PTUR + 2.0-mm-diameter SAF<br>Group III: Mtwo RS<br>Group IV: Mtwo RS+ 2.0-mm-diameter SAF	NO	DW	NM
**23**	Çanakçı et al.	2016	Journal of Endodontics	NM	Mand Premolar	CC between 20°-40°	1 mm short of AF	Reciproc R25 (#25/.08)	CLC	GP+RBS	100% humidity at 37°C for 2 months	Eppendorf Tubes	NM	Group I: PTUR + Reciproc R40(40/.06)<br>Group II: Mtwo RS+ Reciproc R40(40/.06)<br>Group III:	NO	DW	NM

														D-RaCe RS + Reciproc R40(40/.06)<br>Group IV: R-Endo RS + Reciproc R40(40/.06)<br>Group V: Reciproc R25 + Reciproc R40(40/.06)			
**24**	Çiçek et al.	2016	Journal of Conservative Dentistry	NM	Mand Premolar	straight root canals	1 mm short of AF	ProTaper F2	CLC	GP+RBS	100% humidity at 37°C for 2 months	Eppendorf Tubes	NM	Group I: Mtwo RS<br>Group II: Mtwo RS + Mtwo files (30/.05)<br>Group III: PTUR<br>Group IV: PTUR + PTU F3	NO	NM	NM
**101**	Gkampesi et al.	2016	Balkan Journal of Dental Medicine	NM	Single-rooted teeth	CC<5°	1 mm short of AF	ProTaper F3	CLC	GP+CSBS	100% humidity at 37°C for 1 month	Glass vials	NM	Group I: PTUR + PTU F4<br>Group II: Mtwo RS +Mtwo files (40/.06)<br>Group III: R-Endo RS +RevoS files (40/.06)<br>Group IV: GGIII+H files (40/.02)	NO	DW	NM
**9**	Kasam et al.	2016	Journal of Clinical and Diagnostic Research	Y	Mand Premolar	CC<10°	1 mm short of AF	K files #40/.02	CLC	GP+ZOEBS	100% humidity at 37°C for 1 month	Plastic vials	NM	Group I: GG+H files (40/.02) +solvent<br>Group II: Safe Sided H Files (40/.02) +solvent<br>Group III: PTUR+solvent<br>Group IV: Ultrasonic	Y (xylene)	1% NaOCl + saline + 17% EDTA	NM

														Endodontic Retreatment Tip:E-7, E 3~ Max+solvent			
**110**	Pawar et al.	2016	Journal of Conservative Dentistry	NM	Mand Premolar	NM	1 mm short of AF	ProTaper F3	WLC	GP+RBS	100% humidity at 37°C for 1 month	Eppendorf Tubes	NM	Group I: PTUR(control)+solvent<br>Group II: PTUR+PTN X4 +solvent<br>Group III: PTUR+WaveOne Large+solvent<br>Group IV: PTUR+SAF (2 mm) +solvent	Y (Endosolv R)	DW	NM
**25**	Uzunoglu&Türker	2016	European Journal of Dentistry	NM	Mand Premolar	CC<10°	1 mm short of AF	K files #35/.02	CLC	GP+RBS	100% humidity at 37°C for 1 week	Eppendorf Tubes	NM	Group I: Reciproc R40<br>Group II: EdgeFile XR RS(R1-R4)+EdgeFile X3-C4 file (40/.06)<br>Group III: D-RaCe RS+RaCe (40/.04)	NO	DW	NM
**7**	Alfenas et al.	2017	Revista Brasileira de Odontologia	Y	Mand Incisors	CC <5°	1 mm short of AF	K files #35/.02	CLC	GP+RBS	Humid environment at 37°C for 2 weeks	Eppendorf Tubes	NM	Group I: GGII-III+H files+ K files (40/.02)<br>Group II: GGII-III+H files+ K files (40/.02)+ solvent<br>Group III: D-Race RS + BioRace files (BR5 40/.04)<br>Group IV: Mtwo RS+Reciproc R40 (40/.06)	Y (eucalyptol)/NO	DW	Y

**8**	KaşıkçıBilgi et al.	2017	International Endodontic Journal	Y	Max-Mand Molars	CC between 30°-49°	1 mm short of AF	ProTaper Next X2	WVC	GP+RBS	100% humidity at 37°C for 1 month	Glass vials	NM	Group I: GGIII+Reciproc R25+Reciproc R40(40/.06)<br>Group II: PTUR + PTN X4<br>Group III: R-Endo RS + Revo-S AS40<br>Group IV: H-files (40/.02)	NO	DW	NM
**106**	Liu et al.	2017	BMC oral health	Y	Mand First Molars	CC between 25°-35°	1 mm short of AF	ProTaper F1	continuous wave of condensation	GP and sealer (NM)	100% humidity at 37°C for 2 weeks	Centrifuge tubes	NM	Group I: GGI-III+Twisted File (25/.08) at 500 rpm<br>Group II: GGI-III+Twisted File at 1000 rpm<br>Group III: GGI-III+Twisted File at 1500 rpm<br>Group IV: GGI-III+Triple-Flex Files (25/.02)	NO	DW	Y
**35**	Nevares et al.	2017	Iranian Endodontic Journal	Y	Mand Molars	CC 35.5° ± 6.86°	1 mm short of AF	Wave One Small #21/.06	modified hybrid Tagger’s technique	GP+RBS	100% humidity at 37°C for 1 month	Eppendorf Tubes	NM	Group I: Reciproc R25 (25/.08)<br>Group II: PTN X2 (25/.06)	NO	2.5% NaOCl	Y
**113**	Vikram	2017	International Journal of Recent Surgical and Medical Sciences	NM	Mand First Premolar	NM	1 mm short of AF	K files #30/.02 or #40/.02 not clear	CLC	GP+ZOEBS	NM	Eppendorf Tubes	NM	Group I: K files (nm)+solvent<br>Group II: PTUR (nm)+solvent	Y (xylene)	DW	NM
**32**	Yılmaz& Özyürek	2017	Journal of Endodontics	Y	Max Central Insicors	CC<5°	1 mm short of AF	K files #40/.02	WVC	GP+RBS	100% humidity at 37°C for 2 weeks	Eppendorf Tubes	Y	Group I: PTN X5 (50/.06)<br>Group II: TFA ML3 (50/.04)<br>Group III: Reciproc R50 (50/.05)	NO	DW	NM

**36**	Azim et al.	2018	Journal of Endodontics	Y	Mand Incisors	CC<10°	0.5 mm short of AF	Vortex Blue #30/.04	WVC	GP+RBS	100% humidity at 37°C for 1 month (between 30 and 45 days)	Glass vials	NM	Group I: WaveOne Gold Primary file (25/.07)<br>Group II: Hyflex EDM (25/.08)<br>Group III: XP Shaper (27/.01)	NO	DW	Y
**34**	Delai et al.	2018	Brazilian Dental Journal	Y	Max first molars	CC between 20°-40°	1 mm short of AF	Wave One Gold Primary #25/07	CLC	GP+RBS	100% humidity at 37°C for 1 month	Eppendorf Tubes	NM	Group I: LA Axxess #2+ K files (25/.02)<br>Group II: LA Axxess #2+Wave One Gold Primary file (25/.07)<br>Group III: LA Axxess #2+PTUR<br>Group IV: LA Axxess #2+D-RaCe RS	NO	DW	NM
**104**	Jena et al.	2018	Journal of Clinical and Diagnostic Research	NM	Mand Premolar	CC<10°	1 mm short of AF	ProTaper F3	WVC	GP+RBS	100% humidity at 37°C for 10 days	Eppendorf Tubes	NM	Group I: PTUR +solvent<br>Group II: Endostar RS +solvent	Y (RC Solve)	NM	NM
**114**	Pesic et al.	2018	Vojnosanitetski Pregled	NM	Mand Premolar	CC<10°	1 mm short of AF	BioRaCe BR5 #40/.04	CLC	GP+RBS	In saline at 37°C for 3 weeks	Eppendorf Tubes	NM	Group I: GGIV-VI+H files (40/.02)<br>Group II: ProFile (40/.04)<br>Group III: PTUR+PTU F4<br>Group IV: D-RaCe RS+BR5 (40/.04)	NO	3% NaOCl +17% EDTA+DW	NM
**10**	Shivanna et al.	2018	CODS Journal of Dentistry	NM	Mand Premolar	straight canals	1 mm short of AF	ProTaper F3	SC	GP+RBS	In an incubator at 37°C for 1 week	Eppendorf Tubes	NM	Group I: H files<br>Group II: H files + solvent<br>Group III: PTUR<br>Group IV: PTUR +solvent<br>Group V: Mtwo RS	Y (nm)/NO	DW	NM

														Group VI: Mtwo RS +solvent			
**112**	Topçuoğlu et al.	2018	Journal of Investigative and Clinical Dentistry	Y	Mand Premolar	CC<10°	1 mm short of AF	ProTaper F4	CLC or SC or WVC	GP+RBS or GP+CSBS	100% humidity at 37°C for 1 Week	Eppendorf Tubes	NM	Group I: PTUR+ PTU F5+solvent	Y (eucalyptol)	DW	NM
**99**	Balseca et al.	2019	The Open Dentistry Journal	NM	Mand Premolar	NM	NM	ProTaper F3	CLC	GP+CHBS	100% humidity at 37°C for 15 days	Eppendorf Tubes	NM	Group I: PTUR + PTU F4 Group II: Reciproc R25+ R40	NO	DW	NM
**41**	Çanakçı et al.	2019	Nigerian Journal of Clinical Practice	Y	Mand Central Incisors	CC<10°	1 mm short of AF	RevoS SU #25/.06	CLC or WVC	GP+RBS	100% humidity at 37°C for 2 months	Eppendorf Tubes	NM	Group I: PTUR+RevoS AS40 (40/.06)	NO	DW	NM
**96**	Sarıçam et al.	2019	International Dental Research	Y	Mand Premolar	straight roots	1 mm short of AF	K files #30/.02	CLC	GP+RBS	100% humidity at 37°C for 28 days	Eppendorf Tubes	NM	Group I: GGI-III+K files (40/.02)+ solvent Group II: PTUR+PTU F4+solvent	Y (chloroform)	DW	Y
**108**	Aldajani &Mathew	2020	Journal of Clinical Cases and Reports	Y	Mand Premolar	NM	1 mm short of AF	ProTaper Next X3	SC	GP+RBS	100% humidity at 37°C for 1 month	Eppendorf Tubes	NM	Group I: Hyflex EDM files +solvent Group II: ProTaper Gold files +solvent Group III: Hyflex EDM files Group IV: ProTaper Gold files	nm)/NO	NM	NM
**40**	Kamil&Al-Sabawi	2020	Tikrit Journal for Dental Sciences	NM	Mand Premolar	straight root canals	1 mm short of AF	2Shape file #25/.04	continuous wave of condensation or SC	GP+RBS	100% humidity at 37°C for 1 month	Eppendorf Tubes	NM	Group I: D-RaCe RS DR1-DR2 (25/.04) Group II: R-Endo RS R1-R3 (25/.04) Group III: EdgefileXR RS R1-R4 (25/.04)	NO	DW	NM

**26**	Li et al.	2020	The New Armenian Medical Journal	NM	Single-rooted teeth	NM	1 mm short of AF	ProTaper F2	WVC	GP+RBS	100% humidity at 37°C for 10 days	Centrifuge tubes	NM	Group I: GGII-III+H files (30/.02)<br>Group II: GGII-III+H files (30/.02)+desocclusol<br>Group III: GGII-III+H files (30/.02)+eucalyptol<br>Group IV: GGII-III+H files (30/.02)+d-limonene	Y (desocclusol/eucalyptol/d-lionene)/NO	DW	NM
**109**	Mircheska et al.	2020	Balkan Journal of Dental Medicine	NM	NM	NM	1 mm short of AF	K files #30/.02	SC	GP+ZOEBS	in DW at 20-25°C for 3 weeks	Eppendorf Tubes	NM	Group I: H files (nm)<br>Group II: PTUR<br>Group III: ProTaper Gold F5	NO	DW	NM
**39**	Romeiro et al.	2020	Clinical Oral Investigations	Y	Mand Molars	CC between 20°-40°	1 mm short of AF	Reciproc R25 (#25/.08)	SC	GP+RBS or GP+CSBS	100% humidity at 37°C for 1 month	Eppendorf Tubes	Y	Group I: GGII+Reciproc R25+Reciproc R40 (40/.06)<br>Group II: GGII+Reciproc Blue RB25+Reciproc Blue RB40 (40/.06)	NO	2.5% NaOCl+17% EDTA+DW	Y
**30**	Topçuoğlu et al.	2020	International Endodontic Journal	NM	Mand Premolar	CC<10°	1 mm short of AF	Revo-S #40/.06	CLC	GP+RBS	100% humidity at 37°C for 1 week	Eppendorf Tubes	NM	Group I: GGII-III+H files (50/.02)<br>Group II: Reciproc R25+Reciproc R40+Reciproc R50 (50/.05)<br>Group III: PTUR+PTU F5 (50/.05)<br>Group IV: D-RaCe RS+BioRaCe (50/.04)	NO	DW	NM

**94**	Aktemur Türker& Kaşıkçı	2021	Turkish Endodontic Journal	Y	Mand Premolar	straight roots	0.5 mm short of AF	ProTaper Next X3	SC	GP+RBS	100% humidity at 37°C for 1 week	Eppendorf Tubes	NM	Group I: Reciproc R40<br>Group II: Reciproc R40 +XP Finisher<br>Group III: Reciproc R40+XP Finisher R	NO	DW	NM
**95**	AlOmari et al.	2021	International Journal of Dentistry	Y	Mand Premolar	CC<10°	1 mm short of AF	ProTaper Next X2	WVC	GP+RBS	100% humidity at 37°C for 28 days	Eppendorf Tubes	NM	Group I: GGIII-IV+Reciproc R25 (25/.08)<br>Group II: GGIII-IV+XP Shaper (27/.01)	NO	DW	Y
**31**	Dadalti et al.	2021	European Journal of General Dentistry	Y	Mand Premolar	CC<25°	1 mm short of AF	ProTaper F3	WVC	GP+RBS	100% humidity at 37°C for 7 weeks	Eppendorf Tubes	NM	Group I: Mtwo RS<br>Group II: PTUR<br>Group III: Reciproc R25<br>Group IV: K+H files (30/.02)	NO	5.25% NaOCl	NM
**111**	Pirani et al.	2021	International Journal of Dentistry	Y	Single-rooted teeth	CC<20°	NM	HyFlex CM files #25/.04	SC or carrier-based obturators	GP+SBS	100% humidity at 37°C for 1 month	Eppendorf Tubes	NM	Group I: HyFlex EDM orifice opener+Removerins(30/.07) cont rot<br>Group II: HyFlex EDM orifice opener+Removerins(30/.07) jeni mode	NO	DW	NM
**4**	Serefoğlu et al.	2021	Restorative Dentistry & Endodontics	Y	Max first molars	CC <5° and CC between 30°–45°	1 mm short of AF	Wave One Primary #25/.08	SC	GP+RBS	100% humidity at 37°C for 1 month	Glass vials	NM	Group I: H files (40/.02)<br>Group II: R-Endo RS+ Revo-S (40/.06)<br>Group III: Reciproc R25+Reciproc R40<br>Group IV: PTUR+ PTN X4 (40/.06)	NO	DW	NM

**102**	Hassan et al.	2022	European Endodontic Journal	Y	Mand Premolar	CC with 10°	1 mm short of AF	BioRaCe BR4 #35/.04	CLC	GP+RBS	100% humidity at 37°C for 1 week	Eppendorf Tubes	NM	Group I: GGIII-IV+H files+K files (40/.02) Group II: D-RaCe RS+ BR5 (40/.04) Group III: D-RaCe RS+XP Finisher Group IV: D-RaCe RS+XP Finisher R	NO	2.5 % NaOCl	Y
**105**	Karova et al.	2022	Scholars Journal of Dental Sciences	NM	Mand Incisors	CC<5 °	1 mm short of AF	XP-Endo Shaper #30/.04	WVC	GP+RBS	NM	Eppendorf Tubes	NM	Group I: Mtwo RS Group II: D-RaCe RS	NO	DW	NM

***Abbreviations: RT: Retreatment; Y: yes, NM: None Mentioned, Mand: Mandibular, Max: Maxillary, CC: canal curvature, AF: apical foramen, SC: Single-Cone Technique, CLC: c WLC: warm lateral compaction, GP: gutta-percha, RBS: Resin-based sealer, ZOEBS: Zinc-oxide Eugenol- based sealer, CSBS: calcium-silicate based sealer; CHBS: calcium hydrox drills; PTUR: ProTaper Universal Retreatment System, PTU: ProTaper Universal Files, RS: Retreatment System, TFA: Twisted File Adaptive, PTN: ProTaper Next, BR: BioRaCe, SA distilled water; EDTA: Ethylene diamine tetraacetic Acid; NSD: no significant difference**
